# Genetic diversity of Norway spruce ecotypes assessed by GBS-derived SNPs

**DOI:** 10.1038/s41598-021-02545-z

**Published:** 2021-11-30

**Authors:** Jiří Korecký, Jaroslav Čepl, Jan Stejskal, Zuzana Faltinová, Jakub Dvořák, Milan Lstibůrek, Yousry A. El-Kassaby

**Affiliations:** 1 Czech University of Life Sciences Prague, Faculty of Forestry and Wood Sciences, Kamýcká 129, Suchdol, 165 00 Praha, Czech Republic; 2 Department of Forest and Conservation Sciences, Faculty of Forestry, The University of British Columbia, 2424 Main Mall, Vancouver, BC V6T 1Z4 Canada

**Keywords:** Molecular biology, Environmental sciences

## Abstract

We investigated the genetic structure of three phenotypically distinct ecotypic groups of Norway spruce (*Picea abies*) belonging to three elevational classes; namely, low- (*acuminata*), medium- (*europaea*)*,* and high-elevation (*obovata*) form, each represented by 150 trees. After rigorous filtering, we used 1916 Genotyping-by-Sequencing generated SNPs for analysis. Outputs from three multivariate analysis methods (Bayesian clustering algorithm implemented in STRUCTURE, Principal Component Analysis, and the Discriminant Analysis of Principal Components) indicated the presence of a distinct genetic cluster representing the high-elevation ecotypic group. Our findings bring a vital message to forestry practice affirming that artificial transfer of forest reproductive material, especially for stands under harsh climate conditions, should be considered with caution.

## Introduction

As a species of substantial economic and ecological importance, Norway spruce *Picea abies* (L.) Karst, has been the subject of numerous genetic diversity studies utilizing both biochemical and molecular markers^1–8^. Overall, most studies were conducted on the population level and aimed to identify the species extent of genetic differentiation^9–12^.

As genetic markers evolved, the answers on examined hypotheses improved in their precision, starting with allozyme^1–3^, through more informative microsatellite DNA markers^13–16^, to those relying on next-generation sequencing methods which made it possible to address more specific questions such as the impact of evolutionary factors on the genetic makeup of the species^17–22^. Historically, isoenzyme markers showed a low level of inter-population diversity^2,23,24^. Microsatellite markers are frequently used for population-level studies. However, due to their neutral nature, they are not affected by evolutionary forces such as selection and adaptation processes^12,25,26^.

At present, the availability of genomic-based genotyping platforms offers vast opportunities to address specific Norway spruce research questions, such as identifying candidate genes associated with drought response^27^, wood formation^28^, disease resistance^29^, morphological characteristics^30^ or local adaptations^31^.

Despite extensive gene flow and limited genetic differentiation among populations detected with neutral markers, Norway spruce populations possess strong local adaptation in phenological and climate-related traits along latitudinal gradients^32^ such as cold hardiness and bud set^33^, growth cessation^34^, or latitudinal gradient for red/far-red light ratio in connection to local adaptation for shade tolerance^35^. Also, a provenance effect on grown parameters and phenology was shown^31,36^. Only a few studies have paid attention to epigenetic factors in Norway spruce^37,38^ even that they may play an essential role in adaptative responses^39,40^.

The demographic history of Norway spruce has been widely studied, suggested current distribution originated from two main refugia^7,8^. A more recent study showed that the current distribution of Norway spruce genetic diversity is more admixtured due to repeated evolutionary events such as ancient splits, hybridization, and bottlenecks^20^. Norway spruce is a wind-pollinated species characterized by the high gene flow^3,41^. Along with the species outcrossing nature, other factors enhance gene flow level; namely, artificial spreading of spruce outside the natural grown area^42^ and man-influenced forest reproductive material transfer establishing non-autochthonous forests from non-local gene sources^43^.

Norway spruce possess noticeable variability in crown morphology, especially across substantial altitudinal gradients^44^. Despite a relatively continuous variance in crown parameters within stands, three prevalent morphotypes-ecotypic groups, are distinguished^44–46^ along an altitudinal gradient^47^. The low‐elevation ecotype, *acuminata*, is characterized by broad crowns, toothed cone scales, and comb‐like branches. In central Europe, this form occurs predominantly up to 500 m.a.s.l. In contrast, the high-elevation ecotype, *obovata*, typically has a narrow crown, round cone scales, and flat branches (500–1000 m.a.s.l) and the medium‐elevation ecotype, *europaea*, with rhombic toothed cone scales and a mostly conical crown with medium width and brush-type branches that are usually shorter, denser, and down hanging (1000 m.a.s.l up to tree line). It is essential to emphasize that these forms are not exclusively limited to one elevation or latitudinal zone, although any given Norway spruce ecotypic form is usually prevalent within particular stands^44^. Crown shape has been studied with association to altitude^30^; however, with the exception for our previous study utilizing microsatellite markers^16^, there is a paucity of information addressing the underlying genetic causes for the observed architectural differences among these various ecotypes.

Here, we aimed to elucidate the level of genetic differentiation among three phenotypically identified Norway spruce ecotypes determined by their crown morphology and the altitude of their stands using Genotyping-by-Sequencing (GBS) generated SNP data. GBS utilizes restriction enzyme digestion (RE) to focus on sequences in low-copy genomic regions while minimizing reads in the abundant repetitive sequences^48^ present in conifers^49^. By choosing appropriate REs, the repetitive regions of genomes can be avoided, and lower copy regions are targeted with higher efficiency^50^.

Using rigorously filtered data, we applied three widely used multivariate methods for genetic structure analysis. Results provided consistent outputs identifying a group of high-elevation individuals as a genetically distinct cluster. Additionally, we utilized the Redundancy Analysis (RDA^51^), a method that effectively detects even weak, multiloci molecular signatures to identify SNPs with genotype-environment association signals.

## Material and methods

### Study sites and plant samples

We focused on presumably autochthonous and naturally-regenerated stands composed from trees of morphologically predominant morphotypes (ecotypic forms) within the Czech Republic. Per each ecotype, 150 trees were sampled (N = 450). Stands representing the high- (HE), medium- (ME), and low-elevation (LE) ecotypic forms are located in the Giant Mountains National Park, the Jizera Mountains protected area, and the Bohemian Switzerland National Park, respectively (Table 1).

**Table 1:** Norway spruce ecotypes sampling locations and their climatological data.

Ecotypic form	Elevation (m, a.s.l.)	Geographic coordinates	Temperature (°C)	Precipitation (mm)
Low-elevated (*acuminata*)	194–423	50° 52′ 21″ N 14° 17′ 03″ E	8.2	752
Medium-elevatated (*europaea*)	651–857	50° 47′ 56″ N 15° 14′ 18″ E	5.9	821
High-elevated (*obovata*)	1017–1241	Subplot 1 50° 45′ 44.2″ N 15° 30′ 50.0″ E	4.9	946
	1115–1244	Subplot 2 50° 44′ 13″ N 15° 45′ 47″ E		

Average annual air temperature (T), Geographic coordinates are geometrically centered. Climatic values were long-term average measurements^52^.

**Figure 1:**
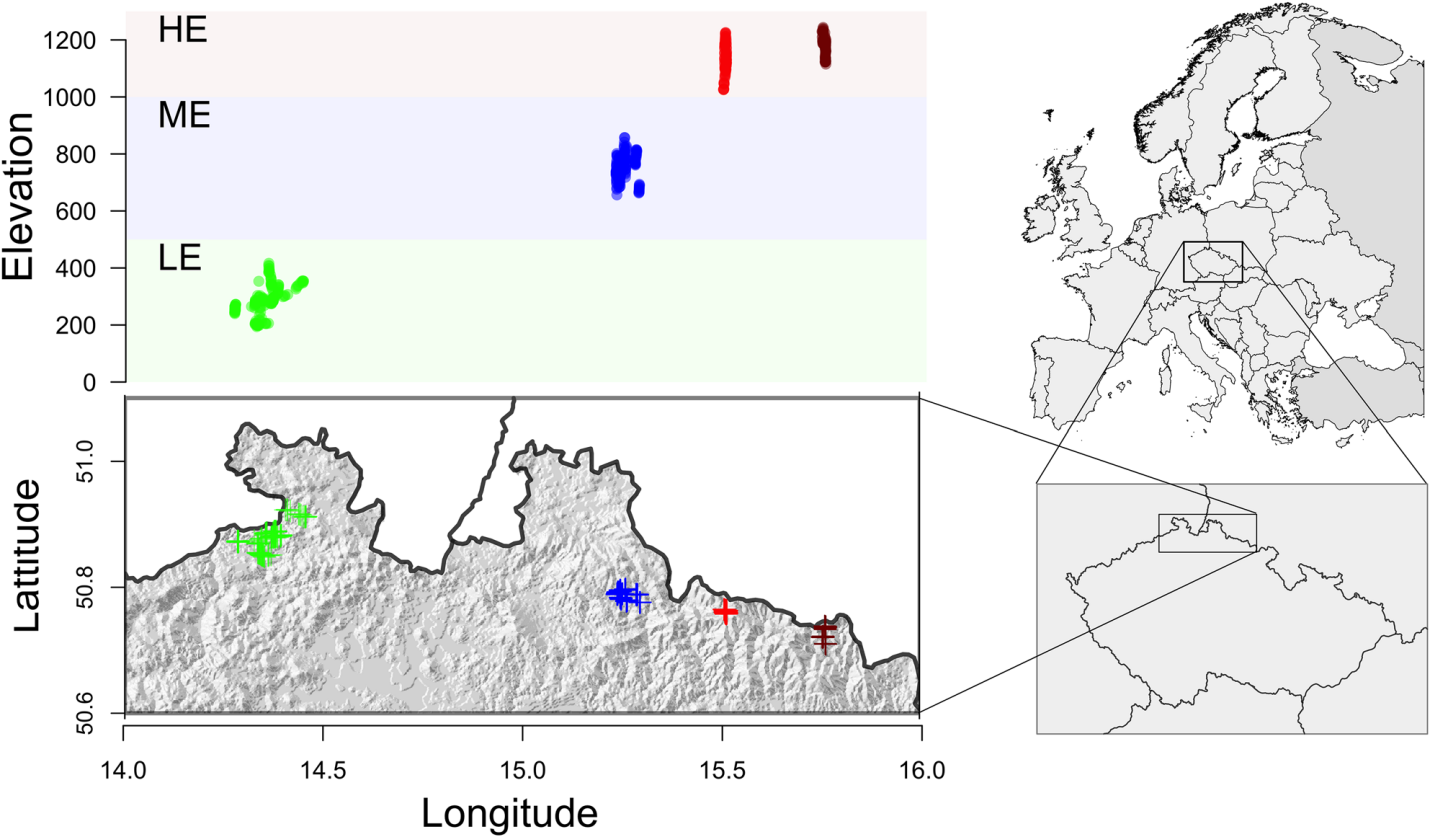
Location of sampling areas. Maps were prepared with R packages raster (v3.5-2)^53^ and RCzechia (v1.8.3)^54^, lines were added using GIPM software^55^. Low-elevation (LE) ecotype (green), medium-elevation (ME) ecotype (blue), and high-elevation (HE) ecotype subplot 1 (red) and subplot 2 (brown).

Generally, sampled trees were > 100-year-old with each tree's GPS coordinates recorded (supplementary Table S2). We targeted mature trees (DBH ≥ 25 cm) of the characteristic crown morphology to their respective ecotypic form (supplementary Figs. S1, S2, and S3). Samples were collected using a hole punch 15 mm in diameter, cutouts from the trunk were kept with silica gel in sealed plastic bags and stored at − 80 °C until further processing. Sampling design consists of transects or clusters with a 25 m as a minimum distance between adjacent trees (see Bínová^16^, for details). HE individuals were sampled across two subplots (Fig. 1).

### DNA extraction

From each sample, approximately 50 mg of cambial and adjacent layers of wood tissue were scratched with a scalpel and frozen in liquid nitrogen, homogenized using a mixer mill MM400 (Retsch, Haan, Germany) for 3 min at 30 Hz. The genomic DNA was extracted by the DNeasy^®^ Plant Mini Kit (Qiagen, Germany) according to the manufacturer's instructions. DNA concentration was measured using a NanoDropTM 2000 spectrophotometer (Thermo Fisher Scientific, Madison, USA). Both intact and *Hind*III (ThermoFisher, USA) digested DNA were run on 0.8% agarose gel to assure DNA quality.Undiluted aliquots of 40 µl DNA (mean concentration 127 ng/µl, 260/280 ratio between 1.47 and 1.91) were placed into 96-well PCR plates (95 samples + one negative control) and shipped under dry ice for analysis.

### Genotyping

DNA samples were digested with the 6-base cutter restriction enzyme EcoT22I. Library and sequencing preparation were done in 96-plex, using 64 bp tag sequences. Sequencing ran on NextSeq 2000 (Illumina, San Diego, CA, USA) platform at the Cornell University sequencing facilities (Ithaca, NY, USA) according to GBS protocol^56^. Reads occurring at a minimal count of three were stored in a FASTQ file format.

### Mapping and SNP calling

FASTQ file was aligned against the *Picea abies* reference genome v1.0., ftp://plantgenie.org/Data/ConGenIE/Picea_abies/v1.0/^57,58^ with Burrows-Wheeler Aligner (BWA, version 0.7.8-r455^59^). Aligned sequence tags were stored in SAM format file and SNP calling was performed using TASSEL-GBS Pipeline (version 3.0.174)^60,61^.

Low-quality SNPs were filtered out by minimum minor allele frequency (MAF) > 0.01 and missing data < 90% and the data were finally converted into Variant Call Format (VCF) file^62^.

### Data filtration

VCF file (raw data) was subjected to a more stringent filtering process before analysis. All filtration steps were performed by VCFtools software (version 0.1.16)^62^ as follow: (1) only biallelic and non-indel SNPs were kept, (2) only loci with no-missing data were kept, (3) MAF was set > 0.05, (4) the Quality value threshold for loci was set ≥ 20, (5) only loci with mean depth values between 10 and 60 were retained, and (6) SNPs within 1000 bp distance were thinned.

### Climatic data

Climatic data were downloaded from WorldClim database (http://www.worldclim.org/bioclim) in 0.5 arc-minute resolution (approx. 1 × 1 km). We utilized *getData("worldclim", var* = *"bio", res* = *0.5, lon* = *15, lat* = *5)* function call from the raster package and assigned each tree stand with given climatic variables. After removing highly correlated variables, seven out of the original 19 climatic variables, were retained for further analysis (Table 2; PCA of selected climatic variables as a Supplement Fig. S4).

**Table 2:** Climatic variables from WorldClim database used for the analysis.

BIO1	Annual mean temperature
BIO4	Temperature seasonality
BIO5	Max temperature of warmest month
BIO6	Min temperature of coldest month
BIO9	Mean temperature of driest quarter
BIO12	Annual precipitation
BIO15	Precipitation seasonality

### Data analysis

Primary data analysis was run with VCF tools. For Principal Component Analysis (PCA), as well as Discriminant Analysis of Principal Components (DAPC), we utilized functions implemented in R package adegenet^63^. For DAPC we used optim.a.score() function to control trade-off between the power of discrimination and over-fitting, to estimate the optimal number of PCs retained (n.pca = 37).

Bayesian clustering was done in STRUCTURE v2.3.4.^64^, the input file was generated with PGDSpider^65^ by VCF file conversion. To detect the most likely number of clusters, we generated K from 1 to 10 (a burn-in period of 10,000 iterations, followed by 100,000 iterations, admixture model scenario) using Evanno's approach^66^.

We used Nei's *G*_*ST*_^67^, Jost's *D*^68^ and Hedrick's *G*_*ST*_^69^ as measures of genetic differentiation among populations. We created *genind* objects for each pairwise comparison with the R package adegenet^63^ and performed 1000 bootstrap samples, with each subpopulation resampled according to its size, with the function *chao_bootstrap()* of the mmod R package^70^. Then, for each set of the permutated datasets, we obtained the observed genetic distance value and its normalized 95% confidence intervals (CI) (i.e., centered on the observed value and corrected with standard deviation across replicates) with the function *summarise_bootstrap()* in mmod package. We considered the genetic differentiation index to be statistically significant if their lower bound of the CI was greater than zero.

We employed the Mantel test from the ade4 package with 100,000 randomizations to estimate the correlation between the genetic distance matrix (*provesti.dist()* from poppr package^71^ and geographical and altitudinal distance matrices. The test uses Pearson's regression coefficient between geographic and genetic distance matrices. The statistics and the test's *p* value correspond to the proportion of times the randomized regression coefficient is equal or greater than the observed one^72^.

Redundancy analysis (RDA) implemented in vegan package^73^ was utilized to identify SNP loci associated with environmental variables for detecting probable adaptive variants^51^. The significance of the global RDA was evaluated by analysis of variance (ANOVA) with 1000 permutations. Then, we identified candidate loci based on locus scores (i.e., the loading of each locus in ordination space) that were ± 3 SD from the mean loading on the first three constrained ordination axes. We recognized the environmental variables exhibiting the strongest associations with each candidate adaptive locus using Pearson's correlation coefficient (*r*). We utilized a permutation test with 100,000 randomizations to estimate *p* value of such correlation and we adjusted α-level by Bonferroni correction (20 candidate SNPs and 7 climate variables) to value 0.00035.

### Sample collection statements

The formal identification of the plant material used in our study was done by Jiri Korecky and confirmed by Jan Stejskal. We declare that the sample collection permissions were obtained from the responsible authorities. LE individuals were sampled in the Bohemian Switzerland National Park under the permission nr. SNPCS 0573/2015, HE individuals were sampled in the Giant Mountains National Park under the permission nr. OSML 38-11/2015, and ME individuals were collected in the Jizera Mountains protected area under the permission nr. LČR 8/2016. Norway spruce is not considered a species at risk of extinction or endangered species. Collection of plant material complied with institutional, national, and international guidelines and legislation. Trees in our study are categorized based on crown morphology (Supplementary Figs. S1, S2, and S3). GPS coordinates of every individual tree are provided in Supplementary Table S2; thus, every tree is traceable. Genomic data are available on-line (https://doi.org/10.6084/m9.figshare.16845445.v1).

## Results

### Genotyping

Four out of the 450 individuals failed in genotyping (one HE, one ME, and two LE), most likely due to the low DNA quality. Among the tested DNA restriction enzymes (EcoT22I, PstI, SbfI/MspI, SbfI/BfaI), EcoT22I, a rare cutter with the potential of good read depth for heterozygotes, was deemed the most appropriate enzyme based on fragment size optimization. There were 96 samples sequenced in one lane turning in approx. 5 M reads per sample. The total sequencing coverage (for 20 Gb Norway spruce genome) has been estimated approximately 0.025×.

### Mapping and SNP calling

The FASTQ file was aligned against the Norway spruce reference genome and from 52,203,003 read tags present, 51.6 and 30.8% were uniquely and multiply aligned, whereas 17.6% tags remain unaligned. Variant calling performed on uniquely aligned tags generated 9,190,592 raw SNPs. These SNPs were filtered by minimum minor allele frequency (MAF) > 0.01 and missing data < 90%, kept 6,970,550 SNPs that were stored in VCF file (mean heterozygosity 0.074 ± 0.012).

### Data filtration

The SNP data underwent a strict filtration in VCFtools software and was reduced from almost 7 million to 1,916 (0.03%) as follows:

1. only biallelic and non-indel SNPs were kept: 6,229,325 SNPs,
2. only loci with no missing data were kept: 36,313 SNPs,
3. MAF was set > 0.05: 16,982 SNPs,
4. the Quality value threshold for loci was set ≥ 20: 16,982 SNPs,
5. only loci with mean depth values between 10 and 60 were retained: 12,824 SNPs,
6. SNPs within 1000 bp distance were thinned: 1,916 SNPs.

The mean observed heterozygosity of the final SNP dataset was 0.271 ± 0.125 (expected heterozygosity 0.237 ± 0.114).

### Data analysis

**Figure 2:**
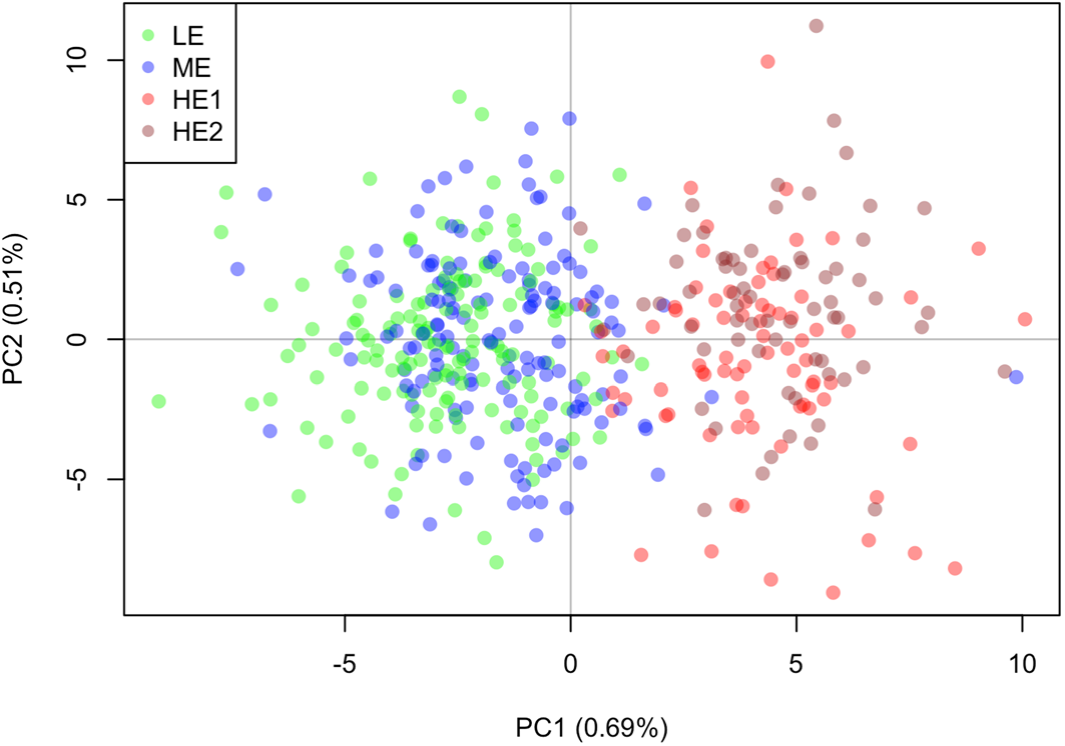
Principal component analysis, PC1 and PC2 are plotted. Each dot represents genotyped individual; explained variance for each PC in the brackets.

The PCA applied on the final dataset (1916 SNPs) separated the high-elevation from the medium and low-elevation ecotypes (Fig. 2). Although the percentage of explained variance is relatively low (PC1 explained 0.69% and PC2 explained 0.51%), the segregation between high-elevation and both ME and LE ecotypes (based on PC2) is visually apparent. It is interesting to note that ME and LE displayed no segregation pattern. Similarly, HE individuals from the two separate locations did not show clustering pattern.

**Figure 3:**
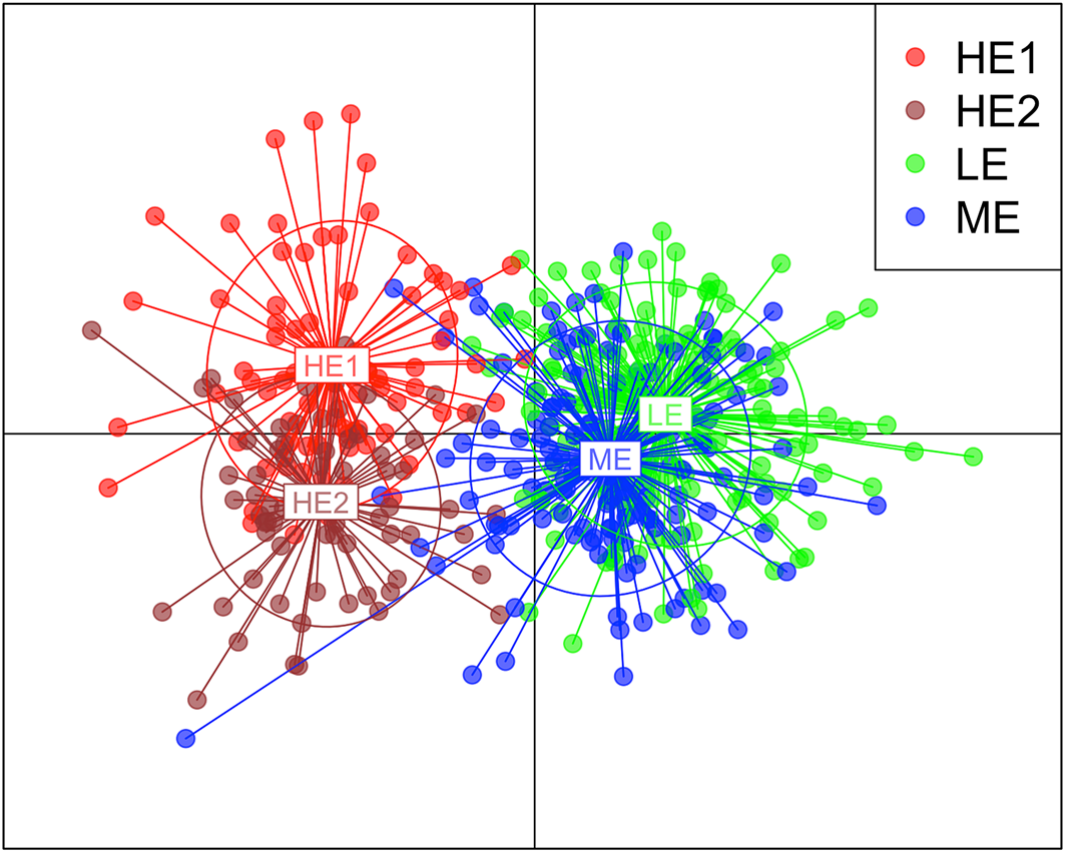
Discriminant analysis of principal components (DAPC) scatterplot showing distinctiveness of the high-elevation (both HE1 and HE2 groups) ecotype in relation to the LE and ME.

For the DAPC analysis, the lowest BIC was not detected (the BIC curve constantly increased along with the increasing number of clusters), likely due to shallow population structure^74^. As a trade-off between the power of discrimination and over-fitting, we used *optim.a.score()* function and retained 37 PCs entering the analysis (n.pca = 37), n.da = 10. Similar to PCA analysis output, the DAPC differentiated the high-elevation ecotype from the low and medium elevation ecotypes. Again and similar to PCA analysis, individuals from both HE subplots overlapped and formed one cluster (Fig. 3).

**Figure 4:**
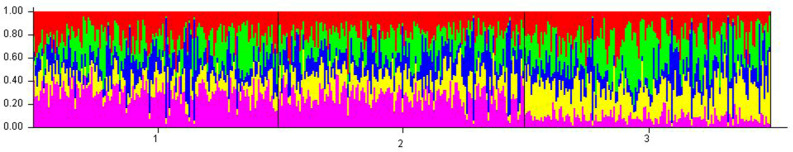
Summary barplot of estimated membership coefficients. Every single vertical line represents one Norway spruce individual in K-colored segments (K = 5) where 1, 2, and 3 represent the LE, ME, and HE ecotypic groups, respectively.

Additionally, clustering using STRUCTURE (K = 5 detected as the most likely number of clusters, a graph for Evanno´s deltaK as Supplementary Fig. S5) showed shallow differentiation among populations; however, a HE Norway spruce ecotype cluster is noticeable (Fig. 4).

**Figure 5:**
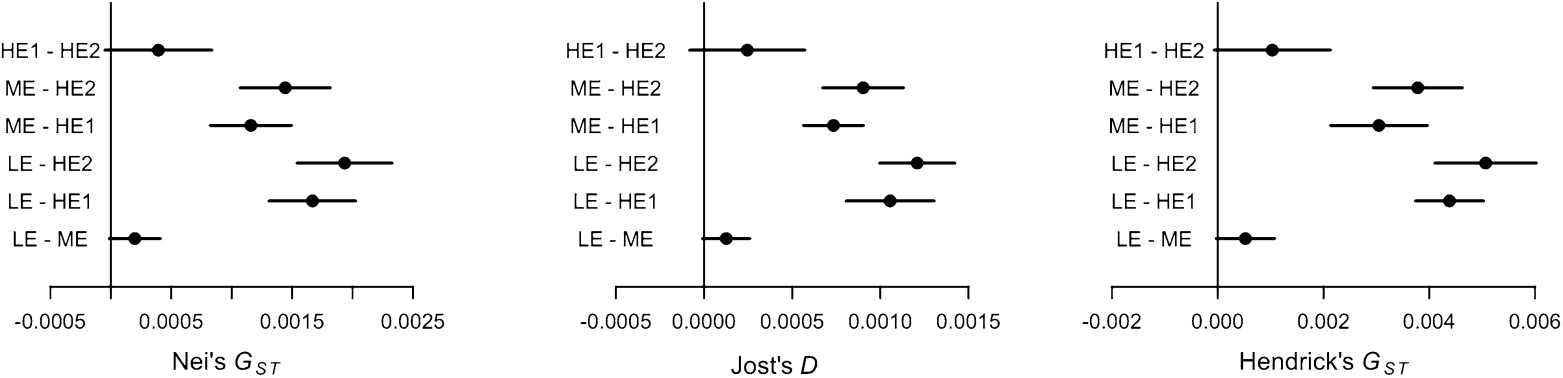
Pairwise genetic differentiation indices among Norway spruce populations. The observed values displayed with normalized 95% confidence intervals.

We estimated Nei's *G*_*ST*_, Hedrick's *G*_*ST*_ and Jost's *D* genetic differentiation indices for each sampled population (Table 3). While the values of genetic differentiation among ecotypes was subtle, significant differences were observed (Table 3, Fig. 5) between the high-elevation and the other two ecotypes with values of one order of magnitude higher than LE–ME or HE1–HE2 pairs (Table 3).

**Table 3:** Overall Nei's *G*_*ST*_, Hedrick's *G*_*ST*_ and Jost's *D* indices for each ecotypic group.

Pop pair	Nei's G_ST_	95% CI	Jost's G_ST_	95% CI	Hendrick's G_ST_	95% CI
LE–ME	0.0002	− 0.00001 to 0.00041	0.00013	− 0.00001 to 0.00026	0.00052	− 0.00003 to 0.00107
LE–HE1	0.00167	0.00131–0.00203	0.00106	0.00081–0.0013	0.00438	0.00374–0.00502
LE–HE2	0.00193	0.00154–0.00233	0.00121	0.001–0.00142	0.00507	0.00411–0.00602
ME–HE1	0.00116	0.00082–0.00149	0.00073	0.00056–0.0009	0.00305	0.00213–0.00396
ME–HE2	0.00144	0.00107–0.00181	0.0009	0.00067–0.00113	0.00378	0.00294–0.00462
HE1–HE2	0.00039	− 0.00005 to 0.00083	0.00025	− 0.00008 to 0.00057	0.00103	− 0.00007 to 0.00213

Genetic differentiation is statistically significant if the lower bound of the CI was greater than zero. Note the HE ecotype is represented by two subplots (HE1 and HE2).

The correlation of genetic distance with the geographical and altitudinal distances evaluated by the Mantel test were 0.062 and 0.068 (*p* < 10^–5^), respectively. While both correlations are low; they were significant, indicating that trees' genetic distance is correlated with altitudinal and geographical distances in an almost similar manner.

**Figure 6:**
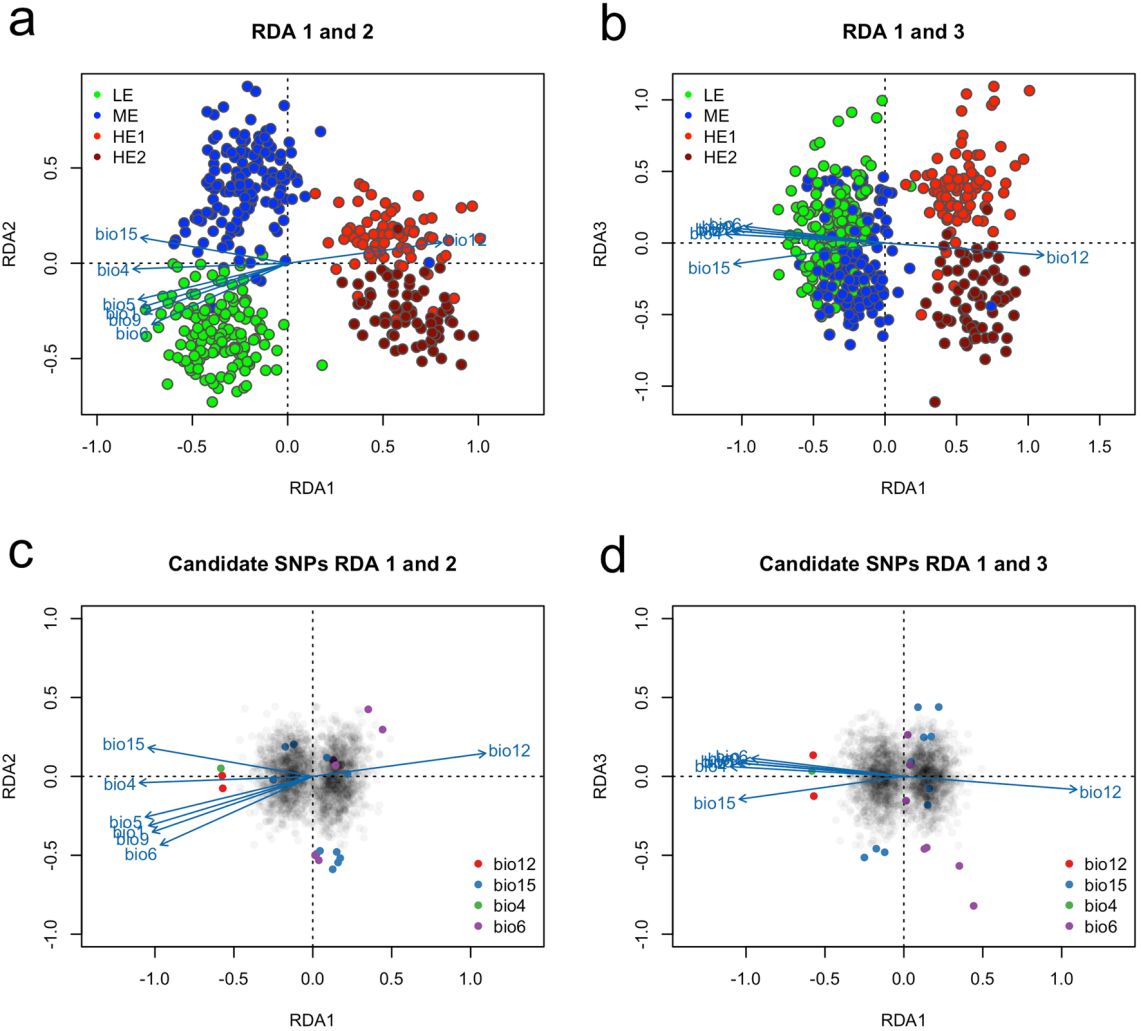
(**a**, **b**) Individuals plotted by their score on the first, second, and third significant axes (**a**) RDA1 and RDA2; b: RDA1 and RDA3), population indicated by color; (**c**, **d**) 1916 SNPs plotted by their score on the first, second, and third significant axes (**c**) RDA1 and RDA2; (**d**) RDA1 and RDA3. Significant outlier SNPs (n = 20) are colored according to their most correlated climate variable (bio4 = temperature seasonality, bio6 = min temperature of coldest month; bio12 = annual precipitation, bio15 = precipitation seasonality).

We used the Redundancy Analysis (RDA) method to select candidate SNPs to depict traces of local adaptations to climatic conditions. The first RDA component separates the high-elevation from medium- and low-elevation populations. In contrast, the second RDA component tends to emphasize differences in low- and medium-elevation populations and the third RDA depicted the difference between HE1 and HE2 populations (Fig. 6a,b). We subsequently detected 20 SNPs, that were ± 3 SD from the mean loading on the first three constrained ordination axes (Fig. 6c,d). Candidate SNPs were correlated with climate variables with 10 SNPs having the highest correlation to bio15 (precipitation seasonality, Pearson's correlation coefficient (*r*) ranging from − 0.05 to 0.13, yet no correlation was statistically significant), 7 SNPs with bio6 (min temperature of the coldest month, *r* ranging from 0.07 to 0.25, 2 correlations were statistically significant), 2 SNPs having the highest correlation with bio12 (annual precipitation, *r* being − 0.23 in both cases, both correlations were statistically significant), and 1 SNP with bio4 (temperature seasonality, *r* being 0.23, statistically significant). See Supplementary Table S1, for details.

## Discussion

### GBS data filtration

Genotyping-by-sequencing is a cost-effective genotyping technique that provides large datasets but is frequently of variable quality. Therefore, initial data filtration is needed, and one should set up the filtration criteria depending on the research question and overall genotyping quality. However, optimal settings are not straightforward and vary widely among studies^75–78^. The only exception is minor allele frequency (MAF) as in similar studies it is conventionally set to 0.05^79–81^. However, settings to different thresholds (0.01 and 0.02) are also reported. Our study inspected a variation attributable to most individuals within the respective group (ecotypic variance), so we set MAF to a 0.05 threshold.

We excluded multiallelic SNPs and indels during the first filtration step, which reduced the dataset to 89.4% of its original size. Further significant reduction occurred when excluded missing data (remaining approximately 0.5% of the original dataset size). Filtering for minor allele frequency additionally further reduced the data set by about one-half. To diminish the effect of linkage disequilibrium^77^, we thinned SNPs within a 1 kbp distance. Being aware of relatively low sequencing depth (mean depth for unfiltered 6.97 M SNPs data set is estimated 3.73), we set the minimum filtration depth for finally selected calling variants to 10, ensuring that only reliable SNPs are kept. We considered this to be the main reason for the low number of SNPs remained after filtering. Although we reduced the primary dataset significantly (preserving approximately 0.03% data out of the primary dataset), the remaining SNPs are robust and reliable.

### Multivariate analyses

Although the interpopulation variation is usually blurred in unsupervised PCA due to summing the variability of both between and within groups^82^, in our data, separation of HE ecotype individuals from LE and ME groups on the first principal component is evident (Fig. 2). We were not able to obtain an unambiguous BIC number with *find.clusters* function;however, that is quite common for populations with shallow population structure^83^. It is noteworthy to emphasize that the direct geographical distance between the medium-elevation sampling area and subplot 1 of the high-elevation stand is approximately the same as between subplot 1 and subplot 2 of HE ecotype (Fig. 1). Based on both PCA and DAPC outputs, it seems that the genetic differentiation is more apparent along the altitudinal rather than the geographical distance. Mantel test is also showing a slightly higher genetic—altitude (0.068) than genetic—geographic (0.062) correlation. However, longitudinal and altitudinal clines are parallel and highly correlated in our setting (Fig. 1), making it difficult to disentangle their effects.

Also in the STRUCTURE barplot, fuzzy yet observable clustering of high-elevation ecotype based on estimated membership coefficients (Fig. 4) is attributable to generally low genetic differentiation indices among defined groups (Table 2, maximum value 0.02). For example, Latch et al.^84^ stated that the software STRUCTURE did not accurately distinguish among populations when the fixation index is lower than 0.03.

We looked for traces of local adaptations toward climatic conditions using RDA, which identified 20 candidate SNPs correlated to climate variables. However, a genome-wide association study conducted with climatic variables did not reveal any significantly associated SNP (not shown). This suggests that the inspected SNPs are not in a strong linkage with genes involved in local adaptation. Given that the Norway spruce genome size is 19.6 Gb^57^, and we have very low-density coverage, with one SNP per ca 800 kb, the likelihood of missing locally adapted regions is relatively high.

### Genetic differentiation among stands

Overall, all genetic differentiation indices (Nei's *G*_*ST*_, Jost's *D,* and Hendrick's *G*_*ST*_) displayed significant differences for the LE–HE and ME–HE ecotypic groups (Fig. 5), whereas HE individuals from the two subplots showed significantly smaller genetic differences. Again, the HE genetic specificity is confirmed for both HE subplots indicating altitudinal rather than geographical differentiation effect. On the other hand, the eastmost HE2 population appears to be slightly more distant to both LE and ME than HE2, again indicating the impact of longitudinal differentiations.

Low genetic differentiation indices values in Norway spruce are common and frequently reported. For example, *G*_*ST*_ values among several German populations ranged from 0.002 to 0.030^85^, *F*_*ST*_ ranged from 0.0004 to 0.0035 among Austrian groups^25^, and from 0.00 to 0.04 among Italian Alpine populations^26^. These results are most likely a reflection of the species' intensive and long-distance pollen gene flow^3,41,86^. However, in contrast with these microsatellite-based studies, our SNP-based analysis might have detected an intrinsic effect of selection in high-elevation ecotype being manifested in our data. The fact that the high elevation samples were collected from two distinct locations provides additional credence to our observation.

Several studies investigated Norway spruce geographical variation along altitudinal gradient based on single nucleotide polymorphisms. Scalfi et al.^87^ with 384 SNPs did not detect any significant *F*_*ST*_ differences for six populations in a 200 m altitudinal gradient. With a set of 175 SNPs, Di Pierro et al.^88^ reported low but significant variation along elevational gradients (*F*_*ST*_ = 0.006). Romšáková et al.^36^ detected considerable differentiation among two altitudinally contrasting populations (700 versus 1450 m a.s.l). Subsequently, Hrivnák et al.^89^ replicated Romšáková's methodology for an altitudinal transect of several populations, reported genetic distinction only for individuals originating from the highest elevation stand (1500 m a.s.l).

It is essential to point out that several studies indicate that most variation in adaptive traits is complex and controlled by many loci of minor effects^31,90^. In our study, we did not detect individual SNPs contributing significantly to genetic grouping, hence we demonstrated the cumulative SNPs effect for ecotypic clustering by multivariate analysis methods.

Additionally, the effects of epigenetic mechanisms^91^ are also attributable to genetic variation, but this effect remained hidden in our study as the GBS approach could not detect them. Although epigenetic modifications are frequently overlooked^50^, it should be more reflected in genetic studies as not all of the variance in the phenotype can be accounted for those coded in DNA sequence^92^. In practice, that can be overcome only by using epigenetics mechanism-sensitive genotyping platforms.

### Pollen transfer and natural barriers

Particularly in coniferous species, pollen gene flow is one of the most critical factors influencing genetic structure^3^. Medium-to-long distance pollen transport is commonly reported^41,93^, with the potential of pollen distribution hundreds of kilometers^3^. This fact reflects the shallow population differentiation revealed by genetic markers^10,11,16^. Conversely, the reproductive success of adjacent potential pollen donors is often limited^3,94^, which has negative consequences on seed orchard production^95,96^. According to Bucci and Vendramin^97^, the increase of the genetic divergence attributable to geographically formed variation is estimated in hundreds of kilometers (approximately 1800 km in Norway spruce). The distance between the sampled populations in our study is limited to circa one hundred kilometers without any natural geographical barriers (e.g., mountain ridges) in between. Therefore, we do not assume the effect of distance and geographical isolation to be factored but most likely selection mechanisms occurring in the HE group. Undoubtedly, also seed dispersal affects population structure, but its effect is more local^98^.

Although we have made every effort to target autochthonous Norway spruce populations, we could not entirely omit the historical effect of the human-mediated stand composition. Jansen et al.^43^ characterized Norway spruce as a highly translocated species for at least three centuries throughout Europe, including the Czech Republic. Sowing of Norway spruce seeds in the Jizera mountains (area of ME ecotype) has been recorded around 1820, but with local provenances. Although since the 1860s, the use of allochthonous forest reproductive material increased, cultivations were performed up to 1000 m a.s.l.^99^. In this regard, the HE ecotype might be considered more likely locally autochthonous. To minimize targeting non-natural regeneration in all three ecotypic groups, we intentionally placed sampling transects on less accessible slopes and rocky outcrops.

### Phenological asynchrony

An additional aspect involved in the genetic differentiation of a given ecotypic group might be connected to reproductive phenology. In oak species, for example, phenological asynchrony driven by microclimatic variability determines the size of acorn crop^100^, in *Eucalyptus* flowering asynchrony build a natural reproductive barrier^101^. In spruce, local synchrony of pollen production and receptivity might promote short-distance pollination and preserve local-adapted genotypes^102^. Skrøppa and Steffenrem^103^ found clinal variation related to mean annual temperature. The intrapopulation variation was considerably larger in low altitude as compared to those from high-altitude populations. Thus, although the mean annual temperature difference of 2.3 °C between LE and ME ecotype is higher than the difference between ME and HE ecotype (1 °C), the narrower biological window of HE pollen receptivity might act as a barrier preventing non-local pollination.

### Natural selection

During the twentieth century, numerous international Norway spruce provenance experiments were established across Europe, known as the Internation Union of Forest Research Organization (IUFRO) provenance trials^104^. Unfortunately, the information about the survival rate of lowland provenances planted in higher altitudes is scarce. Jurásek and Martincová^105^ observed a strong selection effect on Norway spruce seedlings in higher elevations with high mortality levels of lowland-originated seedlings compared to those from higher elevation (1000 m.a.s.l and above). Similar findings were reported by Hrdlička^106^ on a high-elevation testing plot (1070 m.a.s.l.) planted with two seedling sources. After a decade, seedlings originated from lower altitudes (560 m.a.s.l.) exhibit 95% mortality, whereas those from the higher altitude (970 m.a.s.l.) survived seamlessly.

Also in our study, we assume intensive natural selection among high-elevation individuals during the early growing stages as an intrinsic selection mechanism that eliminated unfavorable genotypes for adverse climatic conditions. As we focused on mature trees that persist on the stand despite the ontogenetic selection, we probably largely genotyped individuals that bear distinctive genetic pattern favorable to higher altitudes. Additionally, several ecophysiological studies indicated the resilient specific status of high-elevation ecotype such as enhanced drought tolerance^107^, higher nitrogenous content in needles^108^, and significantly different patterns in dehydrin gene expression^109^.

## Conclusions

The current study aimed to dissect the genetic architecture of three Norway spruce ecotypic forms using SNP markers. We focused on presumably autochthonous stands composed from trees of morphologically predominant morphotypes, a.k.a ecotypes in a limited area of the Czech Republic. Due to such stringent selection criteria, we identified only one experimental area for LE, one area for ME, and two stands for high-elevation ecotypic form.

The data analysis revealed a conspicuous partition into two groups—low- and medium-elevation ecotypes being the one, and the high-elevation forming the second group. Being aware of the experimental limitations that do not allow to distinguish between local adaptation and IBD, we may only hypothesize reasons for such separation.

Although we did not detect any apparent biological (reproductive phenological asynchrony) or geographical (mountain ridges, long-distance separation) barriers, we assume the observed distinctive genetic structure of the high-elevation ecotype is a consequence of a natural selection. Target trees (> 100 years old) have already undergone not only phylogenetic but also ontogenetic selection pressure. Consistently to the long-term observations of forestry practitioners, we assume the intensive ontogenetic selection in a high-elevation ecotypic group promoting local paternal contributors mirrored in the distinctive overall genetic makeup of the extant population. Although our genome coverage was sparse to detect significant genotype-environment associations, we believe we obtained robust outputs in connection to ecotypic distinction. Our findings bring a vital message to forestry management practices. Under the current situation with bark beetle outbreaks across Europe causing increased demand on forest reproductive material for reforestation, some forestry practitioners appeal to relax forest reproductive material transfer rules. That should be considered with caution, especially in locations with extreme climates, as indigenous populations (seed donors) might have previously undergone an intensive selection and adaptation processes and be better adapted to these unfavorable conditions.

## Supplementary Information

Supplementary Information.
