# Supplementary figures and images for: Genetic diversity of Norway spruce ecotypes assessed by GBS-derived SNPs
Source: Sci Rep. 2021 Nov 30;11:23119. doi: 10.1038/s41598-021-02545-z (PMC8632914; doi:10.1038/s41598-021-02545-z)

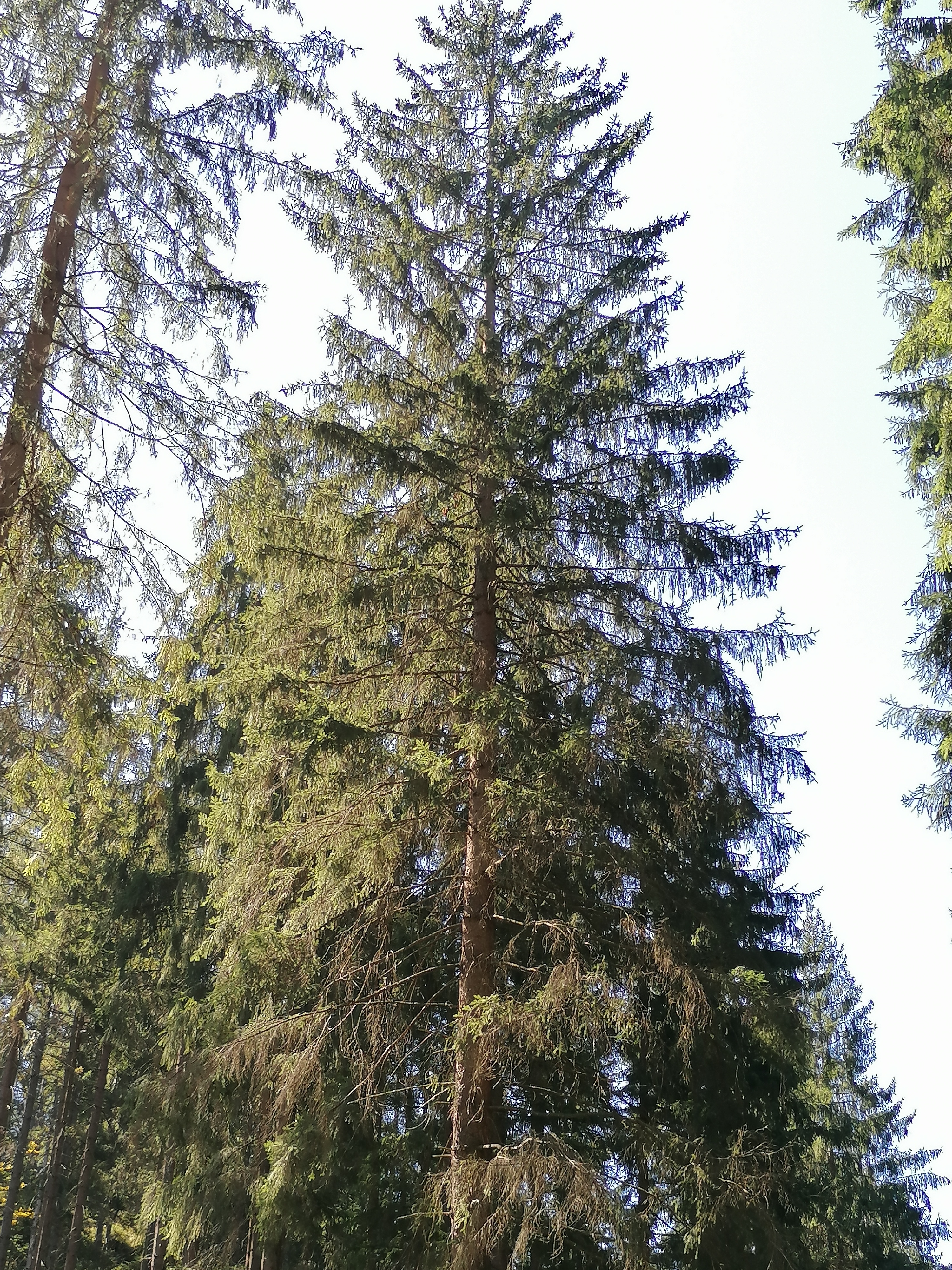

Supplement: Supplementary file 1 — Supplementary Information. [file 41598_2021_2545_MOESM1_ESM.zip › supplementary information/Figure S1 Low-elevation form.jpg]

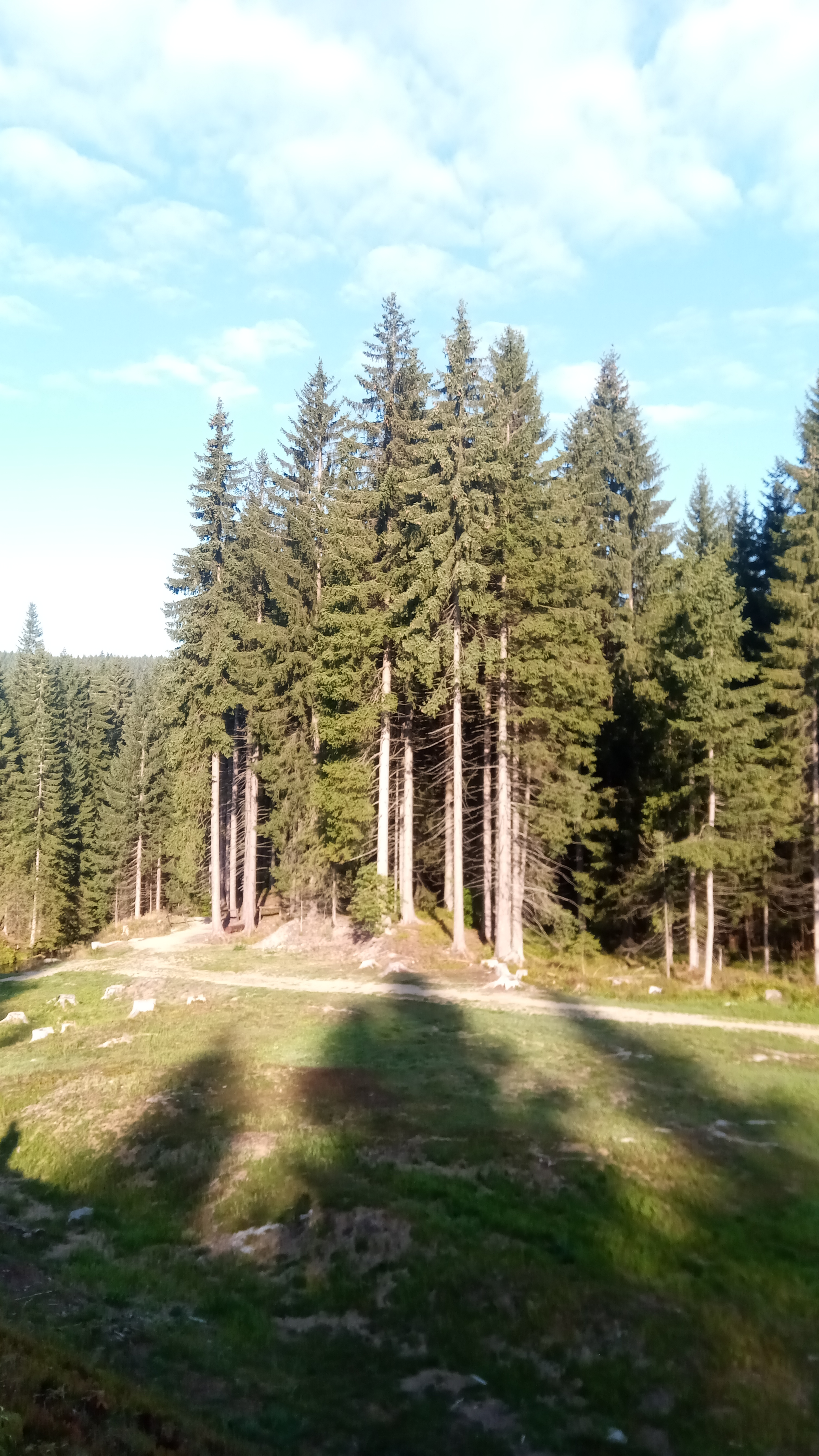

Supplement: Supplementary file 1 — Supplementary Information. [file 41598_2021_2545_MOESM1_ESM.zip › supplementary information/Figure S2 Medium- elevation form.jpg]

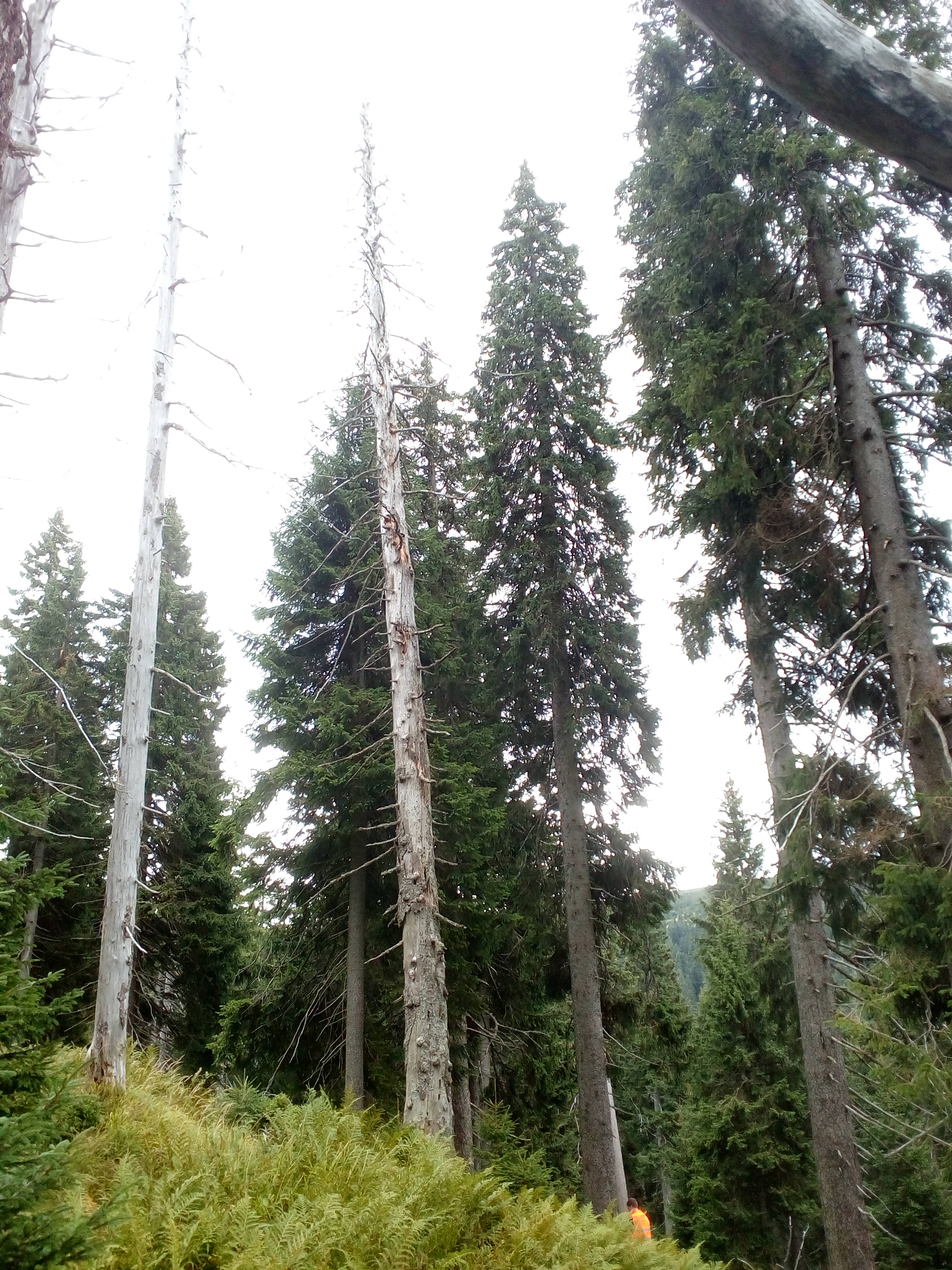

Supplement: Supplementary file 1 — Supplementary Information. [file 41598_2021_2545_MOESM1_ESM.zip › supplementary information/Figure S3 High- elevation form.jpg]

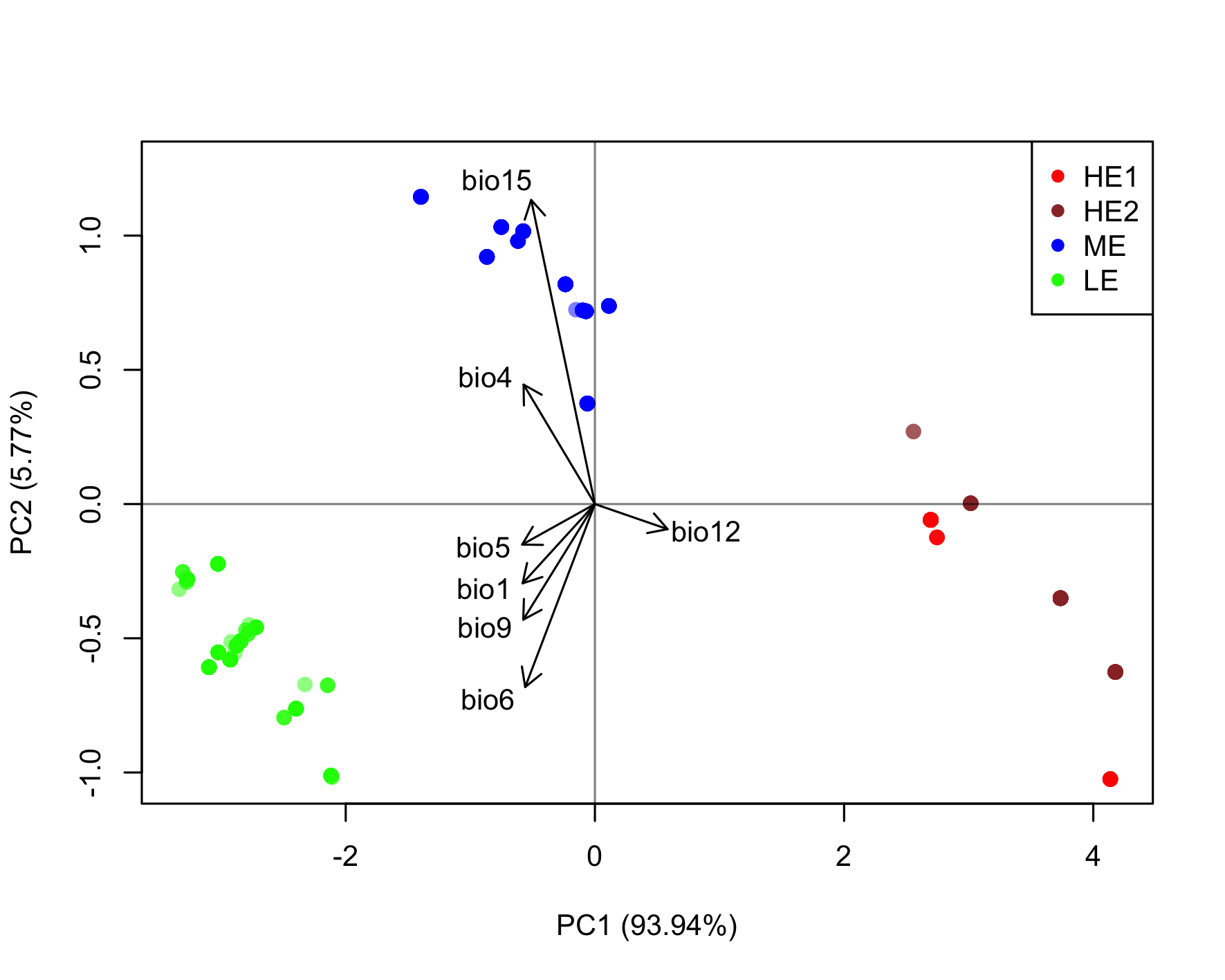

Supplement: Supplementary file 1 — Supplementary Information. [file 41598_2021_2545_MOESM1_ESM.zip › supplementary information/Figure S4_PCA of selected climatic variables.tif]

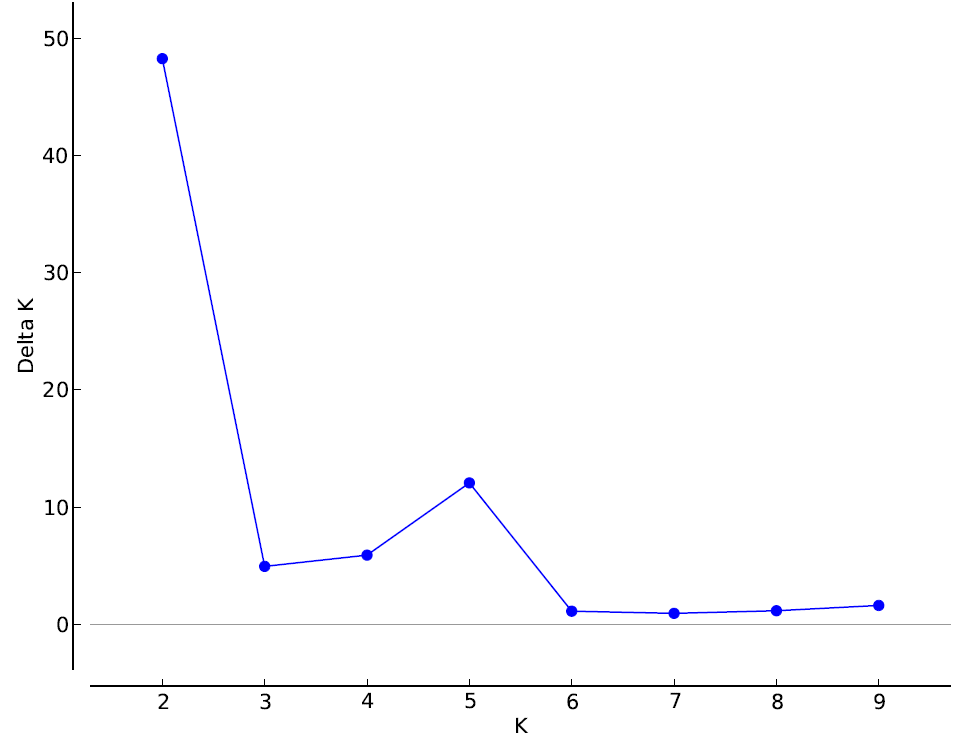

Supplement: Supplementary file 1 — Supplementary Information. [file 41598_2021_2545_MOESM1_ESM.zip › supplementary information/Figure S5_graph for Evannos deltaK.png]
